# Supplementary material for: Are carbon emissions trading and green financial instruments synergistic? -Comprehensive quantitative research based on content analysis
Source: PLoS One. 2024 Mar 7;19(3):e0298601. doi: 10.1371/journal.pone.0298601 (PMC10919601; doi:10.1371/journal.pone.0298601)
Supplement: S1 Data — (ZIP) [file pone.0298601.s001.zip › Data/地级市Z_F工作报告.zip!/║╙─╧/╨φ▓2╩╨/└┤╫╘╣1⁄2╓┌║┼í╛┬φ┐╦╩2╛▌═°í┐_2014.docx]

各位代表：

现在，我代表市人民政府，向大会作工作报告，请予审议，并请各位政协委员和其他列席人员提出意见。

一、2013年工作回顾

2013年，面对错综复杂的形势和艰巨繁重的任务，在省委、省政府和市委的正确领导下，市政府团结带领全市人民，认真贯彻落实党的十八大和十八届二中、三中全会精神，围绕国家粮食生产核心区、中原经济区和郑州航空港经济综合实验区三大战略，坚持“五个着力”，建设“五型许昌”，突出稳增长、调结构、促改革、惠民生，各项工作取得了新的成绩。全市生产总值达到1903亿元，增长10.6%；地方公共财政预算收入108亿元，增长20.1%；规模以上工业增加值1060亿元，增长14.8%；固定资产投资1371亿元，增长23.4%；社会消费品零售总额549亿元，增长13.9%；城镇居民人均可支配收入21717元，增长10.3%；农民人均纯收入11007元，增长12.1%。

一年来，我们突出抓了以下工作：

（一）着力推进项目建设，发展后劲不断增强。大力实施“15512”投资促进计划，150个重点项目完成年度投资559亿元，带动固定资产投资超过1300亿元。黄河集团工业园等69个项目开工建设，森源集团电气产业基地等67个项目竣工投产，在全省产业集聚区项目观摩评比中取得小组第1名。谋划实施了水生态文明城市建设试点、水系连通和50万亩高效节水灌溉三大水利项目，平煤集团6个总投资90亿元项目落户许昌。

（二）着力统筹城乡发展，新型城镇化扎实推进。提升中心城区承载能力，134个城建重点项目完成投资70亿元，49个城中村、棚户区改造项目完成投资58亿元，累计拆迁534万平方米。我市被确定为全国首批、全省唯一的“债贷组合”试点城市。加快许昌城乡一体化示范区建设，路网框架基本形成，配套设施加快完善，商务中心区建设成效明显。推动组团城市发展，中心市区与组团城市快速通道全部建成。30个中心镇建设完成投资25亿元，占年度计划的125.7%。全国文明城市创建工作深入推进，精细化管理不断加强，荣获国家节水型城市、河南省宜居城市称号。

（三）着力调整优化结构，发展方式加快转变。深入实施产业集聚区提升工程、十大产业链发展计划和工业稳增长调结构百日攻坚行动，三大主导、四大特色、五大战略性新兴产业主营业务收入分别达到1938亿元、965亿元、320亿元，在省社科院河南区域工业竞争力综合评价中排名第3位。全面推进现代农业建设，粮食总产达283万吨，新增高标准粮田39万亩、农民合作社394个，省级农业产业化集群达到6个。实施服务业提升计划，县（市）特色商业区全部获批，六大物流园区和十大特色产业物流中心建设顺利，许昌成为全国二级物流园区布局城市、国家智慧城市试点。加快自主创新体系建设，经济技术开发区成为国家科技兴贸创新基地，全市专利申请量和授权量均居全省第3位，连续6次荣获全国科技进步先进市称号。加强环境保护和生态建设，万元生产总值能耗、主要污染物减排完成省定目标。

（四）着力深化改革开放，发展活力更加强劲。扎实推进企事业单位改革改制，20家台账单位改制方案全部报批。大力实施“6116”招商引资行动计划，成功举办了三国文化旅游周、中原花博会等重大招商活动，新签约项目468个，利用省外资金319亿元，增长22.4%，合同引进市外资金1151亿元，41个10亿元以上重大招商项目开工建设。创优金融环境，招商银行入驻许昌，6家企业天交所股权挂牌，新增贷款148亿元、直接融资120亿元。积极推进开放平台建设，建立了公共物流、公共交易、公共技术研发等8个外贸服务平台，海关申建取得阶段性进展，出口总额居全省第2位。

（五）着力保障和改善民生，社会事业全面发展。“十项民生工程”全面完成，全市涉及民生的财政支出达146亿元，增长14.1%，占公共财政预算支出的72%。促进就业创业，新增城镇就业9.2万人、农村劳动力转移就业9.6万人。最低工资、城乡低保、五保供养和企业退休人员养老金等标准进一步提高。城镇居民医保和新农合财政补助标准提高到每人每年280元，35个病种纳入重大疾病保障范围。计划生育奖励优惠政策全面落实。开工建设保障性安居工程12534套，基本建成5233套。市广电大厦、中心医院外科病房大楼等一批社会事业项目竣工投用，新建、改扩建公办幼儿园45所、市区小学6所，改造农村义务教育薄弱学校28所，成功举办市第七届运动会，新增16处全国重点文物保护单位。老龄、妇女儿童、残疾人、慈善等各项社会事业全面进步。

（六）着力促进和谐稳定，法治政府建设得到加强。自觉接受人大、政协监督，办理人大代表议案建议92件、政协委员提案379件。切实转变政府职能，进一步精简压缩行政审批事项，取消和免征行政事业性收费30项。深入推进惩治和预防腐败体系建设，健全了监察、财政、司法、审计等多部门协同监督机制，查处了一批违纪违法案件。扎实推进平安许昌建设，安全生产、食品药品监管、信访等工作扎实有效，保持了社会大局和谐稳定。国防动员、人防、民兵预备役建设和对台工作进一步加强，民族宗教、外事侨务、地方史志、档案、统计、地震、气象等工作都取得了新的成绩。

各位代表，在复杂形势下取得这些成绩实属不易，这是省委、省政府和市委正确领导的结果，是市人大、市政协大力支持的结果，是全市广大干部群众团结拼搏的结果。在此，我代表市人民政府，向全市人民，向人大代表、政协委员，向驻许部队、武警官兵、公安民警，向所有关心、支持许昌发展的各界人士表示衷心的感谢和崇高的敬意！

我们清醒地认识到，全市经济社会发展还面临不少困难和挑战：经济回升向好基础仍不稳固，建材、纺织、电力热力等行业面临较多困难；经济结构性矛盾还比较突出，服务业发展相对滞后，新兴业态、高端业态培育不足；城镇化率偏低，城乡一体化发展水平不高；政府职能转变仍需加快，市场机制仍不完善，社会管理有待改进。对此，我们一定高度重视，认真解决。

二、2014年工作总体要求和主要目标

2014年，我市经济下行压力依然较大，主要矛盾和潜在危机交织，有利条件和不利因素并存，我们既要用好加快发展的有利条件，抓住机遇，乘势而上，又要充分估计形势的严峻性，强化问题意识，清醒应对风险挑战；既要加快发展步伐，尽力而为，能快则快，又要坚持实事求是，稳扎稳打，步步为营。

今年政府工作的总体要求是：全面贯彻党的十八大，十八届二中、三中全会精神，认真落实省委、省政府和市委决策部署，围绕“五个着力”，建设“五型许昌”，把改革创新、扩大开放贯穿于经济社会发展各个领域和环节，稳定经济增长，加快结构调整，深化改革创新，改善人民生活，切实提高发展质量和效益，促进经济持续健康发展、社会和谐稳定。

今年经济社会发展的主要预期目标是：生产总值增长9.5%，地方公共财政预算收入增长11%，工业增加值增长12%，固定资产投资增长21%，社会消费品零售总额增长14%，进出口总额增长10%，实际利用外商直接投资增长10%，城镇居民人均可支配收入、农民人均纯收入分别增长9%，城镇新增就业6.5万人，人口自然增长率控制在6.5‰以内，居民消费价格涨幅3.5%左右，城镇化率提高2个百分点，节能减排完成省定目标。

实现上述目标，着力把握以下几个方面：一是切实把握稳中求进的总基调。统筹稳增长、调结构、抓改革、惠民生、促和谐，调中求进，变中取胜，转中促好，改中激活，巩固经济稳中向好态势。二是切实唱响改革创新的主旋律。把改革贯穿于“五型许昌”建设全过程，用改革的精神、思路、办法破解发展难题，激发市场活力。三是切实找准转型升级的着力点。大力发展三产服务业，加快新型城镇化、新型工业化、新型农业现代化和信息化发展，推进结构调整，转变发展方式，提高质量效益。四是切实强化改善民生的新举措。在经济发展的基础上，更加注重社会建设，扩大公共服务，完善社会管理，促进社会公平正义，推动建设和谐社会。

三、2014年重点工作

按照市委经济工作会议部署，围绕总体目标，今年重点抓好9项工作。

（一）突出项目建设，带动经济平稳增长。项目是带动经济发展的基础与核心，始终把项目作为总抓手，依托项目稳增长、调结构、促改革、惠民生。大力实施“15616”投资促进计划。突出抓好151个重点项目，完成年度投资600亿元以上，拉动全市固定资产投资1600亿元以上。这些重点项目，涵盖工业、服务业、社会事业、交通能源、农林水利、城镇建设等6大领域，特别是服务业、装备制造业、三大水利、“债贷组合”试点等项目，事关全局、事关长远，需要上下一致、齐心协力、狠抓落实。扎实推进重大战略工程。抓住政策机遇，积极推进郑许融合、对接郑州航空港经济综合实验区建设、三大水利项目、棚户区和城中村改造等事关全局的重大战略工程。提升项目运作水平。认真做好征地拆迁、手续办理、资金保障等重点工作，积极创造开工条件。着力完善监管体系，对公共投入项目实施全程动态监管，从机制上保证财政投资高效、公开、透明。提升项目谋划水平，围绕国家政策导向和投资重点，立足产业和资源优势，持续谋划储备一批大项目、好项目。

（二）坚持以人为本，科学推进新型城镇化。城镇化是现代化的必由之路。坚持以人为本、尊重规律、积极稳妥、科学推进，着力提高新型城镇化发展质量。增强中心城市带动能力。实施“1665”行动计划，突出中轴线景观打造及两侧、空铁联运南枢纽、天宝路两侧、城市规划区内水系、三国文化产业园、魏都风情区等6大区域综合开发，加快推进总投资513亿元的163个城建重点项目和51个城中村改造项目，分别完成年度投资83亿元、57亿元。实施城市基础设施普查，完善医院、学校、公交、自行车绿道和文化体育等功能配套，发展城市综合体，让城市更宜居、更宜业。坚持建管并重、服务高效，加强城市精细化、信息化、科学化管理，争创第四届全国文明城市。加快城乡一体化示范区建设。落实“7090”重点项目投资计划，实施总投资320亿元的70个重点项目，完成年度投资90亿元以上。突出基础设施建设，加快芙蓉大道、饮马河连通工程等道路和水系建设。坚持产城融合发展，积极培育现代服务业，加快众信国际大厦、城市文化主题公园等重点项目建设，着力打造商务中心区。推进住宅小区、公租房、配套设施建设，完善基础功能。抓好组团城市建设。突出内涵式发展，有序推进4个县（市）老城改造和新城建设，抓好146个城建重点项目和43个城中村改造项目，分别完成年度投资88亿元、59亿元。突出特色式发展，提高30个中心镇规划建设标准，力争完成年度投资29亿元，努力打造具有历史记忆、文化特色、民俗风貌的美丽城镇。加快城市间、城镇间快速通道建设。推进城乡一体化发展。加快住房保障体系建设，稳步推进城镇基本公共服务常住人口全覆盖，促进农村转移人口落户城市、融入城市。高度重视搬迁群众、失地农民补偿安置、就业、就医、入学和社会保障，有序推进城中村改造安置房屋办理完全产权登记工作。开展“美丽乡村”创建，加强农村水、电、路、气等设施建设，改造农村公路300公里，新建农村户用沼气5000口。加快扶贫开发，完成20个村的整村推进任务，确保2.7万人脱贫。

（三）优化调整结构，提高发展质量效益。没有质量的发展是浪费，没有效益的速度是后退。必须结合不同时期、不同阶段的发展实际，调整优化结构，提高质量效益，推进转型升级。坚持把服务业作为调整结构的重要载体。发展生产性、生活性服务业并重，提升传统服务业、发展现代服务业并举，促进三产服务业发展提速、比重提高、水平提升。加快培育现代物流、信息服务、金融、旅游等高成长性服务业，实施物流示范园区创建工程。积极培育科技研发、工业设计、教育培训、商务服务、健康养老等新兴服务业，支持鄢陵县建设全国生态休闲养生基地，推进禹州市加快发展中医药养生保健产业。改造提升传统服务业，创新商业业态、商业模式和服务产品，发展园区地产、商业地产，加快建设商务中心区和特色商业区，力争新开工项目20个，完成投资50亿元。鼓励大型商贸企业网络化经营，推动房地产和商贸流通等传统服务业转型升级。坚持把信息化作为调整结构的重要抓手。用信息化装备经济发展和社会管理等各行各业，促进产业转型升级和结构调整。以国家智慧城市试点建设为载体，大力发展电子政务和社会领域信息化应用，重点推进无线城市、智慧城市、光网城市、宽带乡村和产业集聚区公共服务信息平台全覆盖工程。坚持把科技创新作为调整结构的重要引擎。深化科技体制改革，加强自主创新体系建设，争创国家创新型试点城市。强化企业主体地位，整合科技资源，实施一批重大科技专项，加快企业研发平台建设，新增高新技术企业8家以上，省级工程技术研究中心3—5个。实施规模以上工业企业消除“零”专利计划，年申请专利4000件以上，新培育知识产权优势企业2—3家。完善科技成果转化、科技创新投融资、科技创新评价和激励等机制，促进产学研结合、科技与金融结合、科技与经济结合，带动产业结构升级。

（四）做大做强工业，提升新型工业化发展水平。新型工业化是稳增长、调结构的重要基础。大力实施“十百千培育工程”，促进工业集约集群集聚、高端高质高效发展。加快产业集聚区建设。继续实施产业集聚区提升工程，突出抓好100个亿元以上产业集群培育项目，带动产业集聚区完成固定资产投资1000亿元，规模以上工业企业主营业务收入超过2400亿元。完善产业聚集区功能性、生产性和生活性基础设施，抓好6个规划调整的产业集聚区新增区域道路管网建设。加快产业转型升级。深入实施十大产业链发展计划，推进100个亿元以上省级工业结构调整项目和10个重点产业集群建设，三大主导、四大特色产业主营业务收入分别突破2000亿元、1000亿元，装备制造业集群主营业务收入突破1000亿元。重点实施30个研发与产业化项目，五大战略性新兴产业主营业务收入突破400亿元。推动企业做大做强。继续实施大企业（集团）培育计划，引导企业以央企改革和淘汰落后产能为契机，沿产业链战略重组，力争主营业务收入超百亿元企业达到8家。加大企业上市培育力度，确保1家企业主板上市，争取1家企业境外上市，6家企业在天交所等区域性股权市场及“新三板”挂牌。落实企业服务长效机制，加强银企对接、产销对接、产业链对接，做好政策咨询服务，帮助企业解决实际问题。

（五）做优做精农业，加快转变农业发展方式。扩内需稳增长，必须加强农业农村工作。围绕稳粮、提效、转型目标，着力发展农业产业化集群，积极推行公司化运作，大力实施农业“三大工程”，不断提升现代农业发展水平。扎实推进高标准粮田“百千万”工程。加快建设国家新增“千亿斤”粮食生产能力规划工程、“五县十乡”万亩高产示范片和50万亩高效节水灌溉示范区，推进中低产田改造和土地综合整治，新增高标准粮田40万亩，粮食总产稳中有增。大力实施现代农业产业化集群培育工程。加快6个省级农业产业化集群建设，新培育省级农业产业化集群2—3个，争创省级示范性农业产业化集群。继续抓好“千场百区”标准化提升工程，加大规模养猪场集约化标准化改造力度，不断提高畜牧业集群发展水平。启动实施都市生态农业发展工程。在中心城区、城乡一体化示范区和有条件的县城周边，以特色种植业、设施园艺业、生态休闲业、农产品加工和流通业为主体，规划建设一批具有综合功能的精准设施农业园区、高新农业科技展示园区、休闲观光农业园区，发展新型都市农业业态。创新农业经营服务方式。有序促进土地流转，推进多种形式规模经营，大力培育专业大户、家庭农场、股份合作或专业合作组织等新型农业经营主体，力争全年新增农民合作社260家、省级龙头企业3—5家，流转土地达到150万亩。完善现代农业服务体系，推动农业科技进步，提高农业物质技术装备水平，大力发展农村金融，建立健全农产品质量安全监管制度和模式。

（六）扩大对外开放，增强经济发展后劲。没有对外开放就没有经济转型。深入开展“对外开放提升年”活动，加快培育市场竞争新优势。全面拓宽开放领域。以国家放宽外资准入为契机，积极开放社会事业、基础设施、基础产业和服务业等领域，吸引国内外大型企业、境内外资本，参与城镇基础设施、医疗卫生、教育科技、健康养老、文化娱乐等领域建设。提升招商引资水平。深入实施“6612”招商引资行动计划，谋划推介600个重点招商项目，推动60个已签约10亿元以上项目落地，力争全年合同引进市外资金突破1200亿元。以港澳台、珠三角、长三角等区域为重点，依托10个产业集聚区，紧盯国内外500强，大力开展产业链招商、专业化集群化招商，努力引进一批具有战略意义的大项目。以现代农业、商贸物流业、医药产业和文化旅游产业为重点，认真筹备三国文化旅游周、花博会、药交会等大型招商活动。借力郑州航空港经济综合实验区，突出完善基础设施、配套关联产业、健全服务功能等重点工作，积极有效利用外资。落实招商引资奖励机制，开展“签约项目落地”专题活动，提高项目履约率、开工率和资金到位率。不断优化开放环境。加快大通关建设，争取许昌海关申建取得实质性进展。推进国家科技兴贸创新基地、国家级外贸转型升级专业型示范基地建设，提升外贸公共服务平台。健全完善招商引资全程服务机制，促进市场公平竞争。

（七）全面深化改革，增强发展内生动力。按照省委、省政府部署，全面深化各项改革，推动重要领域、关键环节改革取得新进展。坚持市场导向、合理配置资源。让市场在资源配置中起决定性作用，着力维护经济环境和公平正义，切实抓好社会保障和社会建设。坚决破除僵化的计划经济思维模式，更加注重发挥市场作用，加快推动教育、卫生、养老、交通、城管、市政等领域发展。积极探索推进人力、资金、资本、土地市场建设，加强市场培育，促进市场成熟。扩大政府购买服务，实行公共服务外包，提高公共服务能力和水平。推进重点领域、关键环节改革。深化国有集体企业改革，大力发展混合所有制经济，加大依法破产、产权出让力度，妥善解决历史遗留和现实问题。加快事业单位分类改革，推进行政职能类和生产经营类事业单位改革。积极推进工商登记制度改革，由先证后照改为先照后证。扎实推进财税体制改革，加强预算绩效和政府债务管理，完善预算制度机制，清理、整合专项转移支付项目，全面落实国家“营改增”、资源税从价计征等政策，优化支出结构，提高资金效益。推进资源性产品价格改革，全面实施居民生活阶梯水价，理顺天然气与可替代能源比价关系，对淘汰类、限制类高耗能企业严格执行差别电价，完成五县（市）农电体制改革。发挥文化资源优势、加快文化改革发展。创新文化体制机制，完善现代公共文化服务体系，推动基层公共文化资源整合、共享和免费开放，提高文化艺术创作和遗产保护水平。深入实施“2348”文化振兴计划，壮大传统优势文化产业，发展动漫游戏、移动多媒体等新兴文化产业，积极培育钧瓷、发制品、生态文化和传媒创意四大文化产业集群，做大做强文化骨干企业，推动文化产业市场化、规模化、专业化、品牌化发展。创新发展理念、破解发展难题。着力增强融资能力，促进资金流动，加强资金监管，提高资金效益，创新金融工具，进一步优化金融生态环境，力争全年社会融资规模达到240亿元。着力提升规划水平，推进各类规划深度融合、科学匹配、衔接套合，让城乡建设多出精品、少留遗憾。着力规范拆迁工作，发动群众、依靠群众，坚持公开、公平、公正，实现依法、阳光、和谐拆迁。着力拓展用地空间，集约节约用地，维护群众土地权益。积极开展人地挂钩和农村集体土地进入市场流转试点，实施城镇低效建设用地再开发专项活动，坚决杜绝“毛地”出让，积极推进农民土地承包经营权、宅基地使用权和集体收益分配权改革，基本完成农村宅基地确权登记发证工作。

（八）建设美丽许昌，推动持续健康发展。全面实施“蓝天工程”、“碧水工程”、“乡村清洁工程”，突出重点领域和行业节能减排，采取硬措施，完成硬任务。抓好污染防治。积极实施蓝天工程，大力推进重点行业脱硫脱硝除尘改造、城市燃煤锅炉改造、机动车尾气治理、城市施工道路扬尘防治、秸秆焚烧整治，努力改善大气环境质量。开展清潩河综合整治和提升工程，推进5个污水处理厂水量提升项目，建设南水北调配套工程。以“清洁家园、清洁水源、清洁田园”为重点，强化土地污染防治，继续开展农村环境综合整治，改善农村环境质量。推进生态建设。加快“五湖”、“四海”、“三川”建设步伐，扎实开展水生态文明城市建设试点工作。加强森林资源培育和保护，全面推进城乡一体绿化，实施矿区生态修复工程，完成林业生态建设造林任务15.2万亩。积极开展生态文明创建，完成2个省级生态乡镇、12个省级生态村创建任务。节约利用资源。全面实施工业、建筑、交通运输等七大节能降耗行动计划，确保全年万元生产总值能耗下降率完成省定目标。实行最严格的水资源管理制度，落实“三条红线”责任目标。持续推进建筑垃圾循环利用、国家节水型城市和“城市矿产”示范基地建设。发挥党政机关表率作用，以创建节约型机关带动节约型社会建设。强化节能减排。加强项目准入管理，严格禁止高污染项目落地，坚决淘汰落后产能，落实节能减排目标责任制、主要污染物排放总量预算管理、生态补偿等制度，积极开展主要污染物排污权有偿使用和交易管理。

（九）保障和改善民生，促进社会和谐稳定。为民发展、为民谋利，是人民政府职责所系、目的所在。突出抓好“十项民生工程”，统筹解决就业、教育、医疗、养老、住房等问题，不断改善群众生活。积极扩大创业就业。以创建国家级创业型城市为载体，实施更加积极的就业政策，加大困难就业群体就业帮扶力度，促进以高校毕业生为重点的青年就业和农村转移劳动力、城镇困难人员、退役军人就业，新增城镇就业6.5万人、农村劳动力转移就业6.5万人，动态消除城镇“零就业”家庭。持续推进全民技能提升工程，完成各类培训20万人次，着力解决就业结构性矛盾。实施“大学生创业引领计划”，举办各类招聘活动，高校应届毕业生综合就业率达到90%。提高社会保障水平。加大社会保险扩面征缴力度，继续提高城乡低保、农村五保和各项社会保险待遇，做好退役军人和随军家属安置。提高新农合财政补助标准，扩大重大疾病保障病种，提高参合群众实际保障水平。落实计划生育奖励扶助政策，全面实施国家免费孕前优生健康检查项目，降低出生缺陷发生风险。完善社会救助体系，加大临时救助，切实保障困难群众尤其是特困群体基本生活底线。健全企业职工工资决定和正常增长机制，推进工资集体协商，构建和谐劳动关系。完善社会养老服务体系，大力发展慈善、残疾人、妇女儿童、红十字等事业。加快实施社会保障一卡通，发放社会保障卡60万张。着力推进面向产业集聚区的公租房建设，新开工保障性安居工程1.2万套，逐步解决务工人员居住难题，改善住房困难群众居住条件。大力发展社会事业。鼓励各类社会资本兴办社会事业，探索政府投资项目所有权、管理权、经营权、使用权相分离的管理模式，激发社会各界参与社会事业的积极性。优先发展教育事业，加快发展学前教育，支持普惠性幼儿园建设，抓好6个市区小学重点项目和76个农村薄弱学校改造项目，缓解城区“入园难”、“大班额”等突出问题。支持普通高中多样化、特色化、优质化发展，保障农民工随迁子女平等享受教育权利。扎实推进“1559”教育信息化工程，探索职业教育产权改革路径，启动实施职业教育攻坚二期工程，推进品牌特色职业学校建设。提高公共医疗卫生服务水平，推进基本公共卫生服务均等化，整顿医疗市场秩序，改善群众就医条件。加快建设东城区三级甲等医院等6个卫生重点项目，支持三级医院与许昌学院合作共建，整合用好区域优质医疗资源。深入实施文化惠民工程，加快建设市科技馆、许昌塔文化博物馆等文化基础设施，扎实推进三国文化产业园等13个文化旅游产业重点项目，办好群众文化活动，满足人民群众文化需求。构建全民健身设施网络，提高竞技体育发展水平，广泛开展全民健身活动。加快气象现代化建设，搞好气象服务。加强社会治理创新。强化安全生产管理，深入实行“签订承诺书、发放明白卡、建立黑名单”制度，抓好重点时段、重点领域、薄弱环节的安全监管和隐患整治，坚决遏制重特大事故发生。重视社会矛盾排查化解，坚持社会稳定风险评估、干部接访、下访、包案等制度，解决好群众的合理诉求。加强基层民主建设，开展第八届村委会换届选举。深化平安许昌建设，加强社会管理重点领域综合治理，推进诚信体系建设，加强网络管理，做好流动人口、特殊人群的管理服务，依法打击违法犯罪活动，提高突发事件应急处置能力。切实加强食品药品安全监管，确保人民群众“舌尖上的安全”。

继续加强国防和民兵预备役建设，促进军民融合发展。支持工会、共青团、妇联、工商联等人民团体发挥桥梁纽带作用。切实做好外事、侨务、港澳、对台工作。继续做好民族、宗教、地方史志、新闻出版、人事、审计、统计、地震、人防、档案、社科研究工作。全面做好第三次全国经济普查工作。

四、以改革精神推进政府自身建设

坚持以转变政府职能为重点，以党风廉政建设为保障，深化行政体制改革，推进行政管理创新，加快建设法治政府和服务型政府。

（一）加快转变政府职能。深入推进简政放权，对保留的行政审批事项实行目录管理，简化审批手续，优化审批流程，严格规范运行，提高服务效率。稳妥推进政府机构改革，强化政府公共服务、市场监管、社会管理、环境保护等职责，推动政府职能加快向创造良好环境、提供优质服务、维护社会公平正义转变。

（二）深入推进依法行政。把政府工作全面纳入法治轨道，用法治思维和方式履行职责。认真办理人大代表议案、建议和政协委员提案，自觉接受市人大及其常委会的法律监督和市政协的民主监督，全面接受社会监督。完善决策咨询、社会听证、重大事项评估等制度，深入推进普法宣传，依法规范行政行为，深入推进政务公开，让权力在阳光下运行。

（三）不断创新工作方法。树立终身学习理念，加强教育培训，全面提升公务员素质，为人民提供优质高效服务。强化责任意识、问题意识，推进观念创新、思路创新、工作创新、管理创新，培育载体、搭建平台、选准抓手、突破瓶颈，不断增强认识问题、分析问题、解决问题的能力。

（四）切实转变工作作风。牢记为民宗旨，深入开展第二批党的群众路线教育实践活动，切实解决“四风”突出问题，提升政府执行力和公信力。厉行勤俭节约，严格控制一般性支出，确保“三公”经费只减不增。健全惩治和预防腐败体系，严厉查处违法违纪案件，把权力关进制度的笼子。大力倡导务实重干，完善目标管理，优化工作流程，提升行政效能，抓好工作落实。

各位代表！建设智慧、创新、美丽、文明、幸福许昌，是全市人民的共同期盼，也是各级政府义不容辞的责任。让我们在省委、省政府和市委的正确领导下，紧紧依靠全市人民，以更加振奋的精神、更加开阔的视野、更加务实的作风，解放思想、开拓创新、万众一心、砥砺奋进，为开创许昌美好未来而努力奋斗！
